# Developing algae as a sustainable food source

**DOI:** 10.3389/fnut.2022.1029841

**Published:** 2023-01-19

**Authors:** Crisandra J. Diaz, Kai J. Douglas, Kalisa Kang, Ashlynn L. Kolarik, Rodeon Malinovski, Yasin Torres-Tiji, João V. Molino, Amr Badary, Stephen P. Mayfield

**Affiliations:** ^1^Mayfield Lab, Division of Biological Sciences, Department of Molecular Biology, University of California, San Diego, San Diego, CA, United States; ^2^California Center for Algae Biotechnology, University of California, San Diego, La Jolla, CA, United States

**Keywords:** microalgae, biotechnology, cyanobacteria, cultivation, breeding, genetic tools for microalgae, essential nutrient, algae

## Abstract

Current agricultural and food production practices are facing extreme stress, posed by climate change and an ever-increasing human population. The pressure to feed nearly 8 billion people while maintaining a minimal impact on the environment has prompted a movement toward new, more sustainable food sources. For thousands of years, both the macro (seaweed and kelp) and micro (unicellular) forms of algae have been cultivated as a food source. Algae have evolved to be highly efficient at resource utilization and have proven to be a viable source of nutritious biomass that could address many of the current food production issues. Particularly for microalgae, studies of their large-scale growth and cultivation come from the biofuel industry; however, this knowledge can be reasonably translated into the production of algae-based food products. The ability of algae to sequester CO_2_ lends to its sustainability by helping to reduce the carbon footprint of its production. Additionally, algae can be produced on non-arable land using non-potable water (including brackish or seawater), which allows them to complement rather than compete with traditional agriculture. Algae inherently have the desired qualities of a sustainable food source because they produce highly digestible proteins, lipids, and carbohydrates, and are rich in essential fatty acids, vitamins, and minerals. Although algae have yet to be fully domesticated as food sources, a variety of cultivation and breeding tools exist that can be built upon to allow for the increased productivity and enhanced nutritional and organoleptic qualities that will be required to bring algae to mainstream utilization. Here we will focus on microalgae and cyanobacteria to highlight the current advancements that will expand the variety of algae-based nutritional sources, as well as outline various challenges between current biomass production and large-scale economic algae production for the food market.

## 1 Introduction

As we begin to reach the limits of our food production systems, one out of every nine people in the world are already suffering from malnutrition, and new food options must be explored to face this rising problem (1). Food insecurity is one of the largest threats of the 21st century and is primed to worsen as climate change and population growth continue to push the limits of our already strained food production systems (1). The production of new food sources that are nutritional and whose production and cultivation minimally impact the environment are needed immediately to compensate for the detrimental byproducts of current agricultural practices.

Recent data has shown there has been an increase in public awareness and a shift in public perception regarding the environmental impacts of the agribusiness industry (2). Public awareness can foster mobilization of international organizations such as OECD, which developed recommendations to the member countries on how to meet sustainability and productivity growth goals (3). Additionally, the impacts of the COVID-19 pandemic have also increased our awareness of the requirement to reduce environmental emissions while simultaneously raising concerns of zoonotic disease transmission from wild meat sources (2). This culmination of environmental, health, and ethical concerns have been the driving forces behind the effort to replace traditionally animal meat-based food products with plant-based alternatives. This consumer shift has expanded the market for vegetarian foods with plant-based meat sales increasing 18% from 2018 to 2019, and is anticipated to have a continual growth rate of 11% per year over the next decade (4).

In terms of environmental impact, meat consumption requires intense land and water usage, and emits significant amounts of greenhouse gasses (GHGs) (5). Unfortunately, GHG emissions are not the only issue posed by today’s agriculture practices. Traditional agricultural systems also contribute to topsoil erosion, abuse of water resources, and nutrient pollution of surface and groundwater (6). Environmental stability of the planet will require the implementation of more efficient and sustainable food production systems; ones that not only provide food for future generations, but also help to mitigate the effects of climate change. When implementing designs for a new, sustainable food source, the usage of arable land, freshwater, and the effects of this new food production on biodiversity must be taken into consideration (7). The use of algae as a future food source has the capacity to address many of these issues.

Algae are aquatic photosynthetic organisms that grow by consuming carbon dioxide, light, and nutrients, and include organisms that range in diversity from giant kelp and seaweed to microscopic single-cellular algae. Despite being strictly defined as eukaryotic aquatic photosynthetic organisms; we will extend the term “algae” to include the prokaryotic cyanobacteria. Here we will focus on the prospective cultivation of microalgae, more specifically (green, red, and brown microalgae), diatoms, and cyanobacteria (blue-green algae). For simplicity, the term “algae” shall refer inclusively to all of these, unless specifically stated otherwise.

Many species of both prokaryotic and eukaryotic algae have natural features that make them desirable as a food source for human consumption (8). Algae are capable of fast and cost-effective photosynthetic growth, have been shown to have positive effects on human health, and have a robust set of tools that can lead to domestication and biomass improvements (9–11). Various species of algae have members that grow relatively quickly and to high biomass concentrations. A 2014 study found that algae can annually produce 167 times more useful biomass than corn when using the same amount of land (12). When producing food for a large population, impressive production figures such as these are key drivers of the overall efficiency and effectiveness of algae as a human food source.

Besides their remarkable yields, algae boast an equally superb nutrient composition that uniquely suits the human diet. Even in small amounts, algae have great potential as dietary supplements that can provide essential macronutrients such as amino acids and fatty acids (8, 13, 14). Multiple algae approved for human consumption have a dry biomass protein content that can range from 27 to 70% protein content (15, 16). As the primary producers of omega-3 fatty acids, such as the essential docosahexaenoic acid (DHA) and eicosapentaenoic acid (EPA), algae are an excellent source from which these products can be derived (16–18). Similar to land plants, algae also provide a rich source of micronutrients such as vitamins and minerals (19). The combination of high protein content, production of essential lipids and fatty acids, and the presence of important vitamins, make algae an ideal candidate for expansion into the human diet.

**FIGURE 1:**
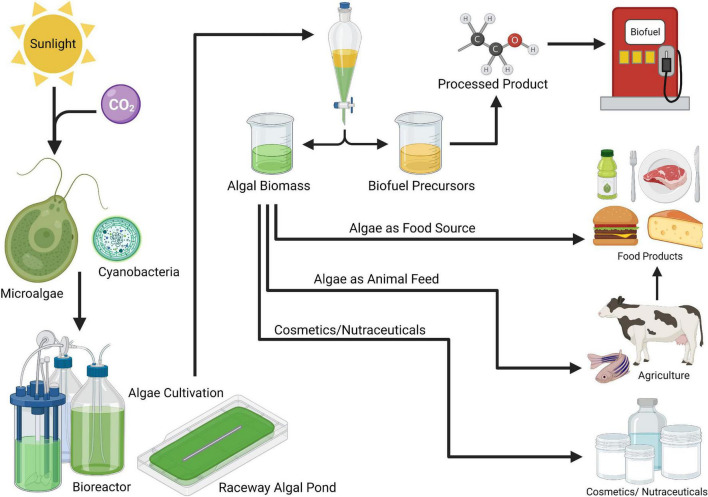
The versatile markets for algae products (Created with Biorender.com).

Algae are the most diverse organisms on the planet, making them excellent candidates for engineering new food products, or emulating existing animal products to meet a variety of nutritional, environmental, and production needs (Figure 1) (20). Some algal species are capable of heterotrophic growth, which further enhances the capability to culture them in regions where outdoor cultivation is not ideal. Heterotrophic growth is generally used for products that require high productivity yields or extremely high product purity (18, 21, 22). Owing to their unparalleled growth efficiency, diverse morphology, and nutritional composition, these single-celled organisms can become a crucial component of feeding future generations of humans.

Currently, lab scale data on algae’s potential as a safe and functional food product are promising, but studies done on agricultural scale production are still required to compare the costs between algae and staple crops (13). This is a major barrier to large-scale adoption of algae as human food or animal feed. Because there is a lack of studies that detail the production costs of algae as food, a discussion about the costs for algae production for biofuels can be used to estimate the costs of producing food from algae. With biofuels, higher operational and production costs were significant obstacles for commercialization, specifically when compared to the costs to produce fuel from fossil fuel sources (23, 24). However, this may not be the case when comparing algae production cost to food cost, as described below.

These economic challenges can be overcome by additional improvements in microalgae strains, improved production processes, and economies of scale as demand increases; but the final unlock will come with the widespread social acceptance toward consumption of algae as an everyday food (25, 26). Changing the perspective on consuming algae is achievable. A case study conducted in Spain found that consumers considered algae to be a “sustainable and environmentally friendly, nutritious and healthy, and safe” food (25). Increasing awareness surrounding algae may encourage more consumers to adopt new eating habits by incorporating algae into their diets, and this increased consumption will help drive down prices as economies of scale begin to kick in.

## 2 Algae, fuel, and food

### 2.1 The many faces of algae cultivation

Algae have been cultivated as a food source for thousands of years; however, an expansion into larger scale algal cultivation was prompted by the biofuel industry, based on the potential of algae to become a highly sustainable biofuel source (8, 27, 28). The ability of algae to efficiently sequester CO_2_ into energy-rich lipids was a driving force for algae biofuel production to gain popularity as an alternative to fossil fuels, which are a major contributor to climate change (29). Other sustainable sources of biofuels require greater energy input to convert biomass into biofuels, making these sources less environmentally favorable (30). Algae biomass production for food or feed will need to undergo different downstream processing compared to the biomass used for biofuel, but the underlying biomass production will be the same (31). After harvesting, algae cells are either dried to be used as food supplements directly, or can undergo further processing for protein, lipid, or carbohydrate extraction. From this point, various downstream products can be produced depending on which macro and micronutrients they possess (31). The desirable molecules for food are very different from the ones used for biofuel applications. For example, longer polyunsaturated fatty acids are desirable for nutritional supplementation, while shorter, more saturated fatty acids are generally preferred for fuel production (32). In all, the success of the algae biofuel industry provided proof of concept for sustainable algae cultivation which can be reasonably translated to the production of microalgae-based food products (18, 33).

### 2.2 Cyanobacteria: Background and biofuel production

Cyanobacteria possess many of the same characteristics as other algae that make them attractive for the production of sustainable products, as they do not require arable land and can grow faster than all terrestrial plants (34). Additionally, several strains of cyanobacteria have the ability to fix atmospheric nitrogen, which is a significant benefit as nitrogen fertilizers are mainly derived from fossil fuel via the Haber-Bosch process (35). Several cyanobacterial species are already in use as biofertilizers, and some are also capable of degrading pollutants (36). Cyanobacteria can also be extremophiles, which allows them to be grown in outdoor ponds at a large scale, in conditions such as high salt, or high pH, which lowers the risk of contamination (36, 37). Marine cyanobacteria are species that are grown in sea water, which eliminates the need to use freshwater for their cultivation (17, 38). Various cyanobacterial species have already been engineered to produce several biofuel related substances including ethanol, isobutyraldehyde, butanol, ethylene, and isoprene, demonstrating their ability to produce a range of compounds (34). Glycogen, a major product of many cyanobacteria can be fermented into ethanol, which can be used as a biofuel, or as a chemical feedstock to make a variety of other compounds (17, 39, 40). Badary et al. (39) showed a unique genetic regulation of glycogen synthesis demonstrated by the marine cyanobacterial strain, *Synechococcus* sp. NKBG 15041c. This regulation is not found in other cyanobacterial species where there is an increase in transcriptional levels of glycogen anabolic genes (glycogen synthase) in response to nitrogen depletion, leading to increased production over other cyanobacterial strains. The overexpression of two glycogen anabolic genes was shown to enhance glycogen accumulation in cyanobacteria (41).

### 2.3 Bridging the divide between biofuels and food with algae biotechnology

Rafa et al. (24) conducted a “techno-economic analysis integrated with models and parameters” to investigate the costs of lipid production in microalgae species *Chlorella vulgaris*, *Tetraselmis suecica*, and *Nannochloropsis* sp. The commercial viability of microalgae-based biofuels depends on outdoor large-scale cultivation, which can occur in open raceway ponds (ORPs) (24). For an open pond system the costs of equipment is only $0.1/m^3^, while nutrients like nitrogen ($0.407/kg), and phosphorous ($0.442/kg) are a much more significant cost (24). Because atmospheric CO_2_ is generally not sufficient to support high microalgal growth rates, additional CO_2_ is often used for full-scale biofuel production (42). Therefore, the price of CO_2_ supply equipment and CO_2_ gas also need to be considered. Adopting algae as food will entail similar cost challenges to that of algae-derived biofuels. In the study by Rafa et al. (24), the costs of biomass harvesting, culture dewatering, and pre-treatment are thoroughly discussed. Based on this techno-economic analysis, it is evident that the cost of lipid extraction from microalgae to produce biofuels at a commercial level is the most expensive part of the process. Although lipids are also a major nutritional molecule found in microalgae, they do not need to be extracted, but rather are consumed in the whole algae, making this expense unnecessary for algae as food. From these biofuel analyses, one can extrapolate that additional technological innovation at a large scale will be necessary in order to bring production costs for microalgae as food to that of current conventional crops. However, with the added nutritional benefits of algae compared to traditional crops, some higher cost can be tolerated.

Although algae are abundantly capable of producing sustainable biofuels, the point remains that other forms of renewable energy (solar, wind, etc.) are more economically competitive for the current energy market (43). This makes the transition to sustainable food sources a highly attractive option for algae production, since food will always need to be obtained from a biological source, and products produced from algae will be able to compete at market prices (8). Although more efficient algal strains are still being developed, simulation models have predicted that existing algae strains have the potential to replace 25% of European protein consumption and 50% of the total vegetable oil consumption when grown on available land that is not presently used for traditional crops (7). Improvements in algal growth rate and biomass quality can increase the sustainability of algae by reducing the resources, land, water, and energy presently used for their cultivation. In order to decrease costs, improvements in harvesting, downstream processing, and growth media recycling, have already been developed that can be built upon to improve the overall economic viability of microalgal production (29, 32, 44, 45). The cost of algae produced as food is still higher than current market prices for commodity crops like soybeans; however, algae-based food products remain an attractive option to pursue due to their potential to create higher quality nutrients. This is compounded by a low potential target price for algae production and the ever-increasing demands for commodity foods that are unlikely to be met by soy and other traditional crops (7, 32).

### 2.4 Nutritional value of algae

The three basic components of any food are proteins, lipids, and carbohydrates. In general, the worldwide agricultural landscape has an abundance of carbohydrates, as these are the main components of corn, rice, cassava roots, and many other grains that are the major crops of the world (46). Proteins and essential lipids are scarcer, with soybeans being the world’s major crop for these two components, of which the protein portion of their biomass is in greatest demand (47). Fortunately, algae accumulate large amounts of both proteins and lipids that are both highly digestible and nutritionally well-balanced (Table 1.2). Indeed, algae is already used as a food additive to enhance the nutritional quality cereal-based products, dairy, and even meat-based products, just to name a few (48). The protein content of algae is particularly impressive, as it contains all of the essential amino acids required for the human diet with comparable amounts (if not higher) to those of traditional crops (Table 1.2). A thorough review of algae complete nutritional content has been recently covered by Torres-Tiji et al. (18), which is summarized in Tables 1.1, 1.2 below.

**TABLE 1.1:** Nutrient content comparison between algae and highly produced traditional crops.

Nutrient	Spirulina	*Chlorella vulgaris*	Soybeans, mature seeds, raw	Wheat enriched, unbleached	Flour, rice, white, unenriched	Corn flour, yellow, fine meal, enriched
Lipid	2.2 g/100 g (16)	0 g/100 g (70)	19.9 g/100 g (158)	1.48 g/100 g (159)	1.3 g/100 g (160)	1.74 g/100 g (70)
Protein	63 g/100 g (16)	60 g/100 g (70)	36.5 g/100 g (158)	13.1 g (70)	6.94 g/100 g (70)	6.2 g/100 g (70)
Carbohydrate	22 g/100 g (16)	40 g/100 g (70)	30.2 g/100 g (158)	73.2 g/100 g (70)	79.8 g/100 g (70)	80.8 g/100 g (70)
Iron	58 mg/100 g (16)	240 mg/100 mg (70)	15.7 g/100 g (158)	5.41 mg/100 g (70)	0.22 mg/100 g (70)	4.44 mg/100 g (70)
Calcium	1,000 mg/100 g (16)	33 mg/100 mg (70)	277 mg/100 g (158)	21 mg/100 g (70)	6 mg/100 g (70)	0 mg/100 g (70)
Magnesium	400 mg/100 g (16)	∼274 mg/100 g dry weight [average from Table 4 in Watanabe et al. (161)]	280 mg/100 g (158)	33.3 mg/100 g (70)	22.9 mg/100 g (70)	0.172 mg/100 g (70)
Zinc	3 mg/100 g (16)	∼1.5 mg/100 g dry weight [average from Table 4 in Watanabe et al. (161)]	4.89 g/100 g (158)	0.9 mg/100 g (70)	1.19 mg/100 g (70)	0.62 mg/100 g (70)
Potassium	1.4 mg/100 g (16)	0 mg/100 mg (161)	1,800 mg/100 g (158)	135 mg/100 g (70)	75 mg/100 g (70)	144 mg/100 g (70)

**TABLE 1.2:** Amino acid content comparison between human dietary requirements, algae, and highly produced traditional crops.

Amino acid	Human requirement	*Dunaliella bardawil*	*Spirulina*	*Chlorella vulgaris*	Soybean	Chickpea	Wheat	Rice (Japonica)
Histidine	10	1.8	1.8–2.2	2	2.6	0.214	1.8	0.9
Isoleucine	20	4.2	6.0–6.7	3.8	5.3	0.379	3	0.8
Leucine	39	11	8.0–8.9	8.8	7.7	0.635	6.8	1.9
Lysine	30	7	4.6–4.8	8.4	6.4	0.521	2.8	0.4
Methionine	10.4	2.3	1.4–2.5	2.2	1.3	0.087	1.9	0.6
Phenylalanine	25	5.8	4.9–5.3	5	5	0.476	4.4	0.7
Threonine	15	5.4	4.6–6.2	4.8	4	0.364	2.6	0.5
Tryptophan	4	0.7	1.4	2.1	1.4	0.037	1.3	0.9
Valine	26	5.8	6.5–7.1	5.5	5.3	0.379	4.5	1.9
References	(162)	(18)	(1)	(163)	(164)	(165)	(166)	(167)

Another notable nutritional characteristic of microalgae is its potential to be a source of omega-3 fatty acids. Omega-3 fatty acids are polyunsaturated fatty acids and provide several vital health functions for eukaryotic organisms (49). Omega-3 fatty acids are especially essential for the human diet as the human body is not able to synthesize them on its own at a fast enough rate to satisfy metabolic needs (50). EPA and DHA are the most beneficial omega-3 fatty acids, and their consumption has been shown to combat cardiovascular, nervous system, and inflammatory conditions (51). Omega-3 fatty acids additionally aid brain function and are necessary for the healthy development of a fetal brain (49). The current dominant source of dietary omega-3 fatty acids come from fish such as salmon, mullet, and mackerel (51), which are in the end of the food chain that started with the primary producers, algae. However, extreme overfishing has made it necessary to look elsewhere for sources of omega-3 fatty acids. Although omega-3 fatty acids can also be found in fungi and plants, the need for an organic carbon source and arable lands respectively decreases the viability of these as sustainable alternative sources (49).

Microalgae presents as a sustainable source of omega-3 fatty acids and has been vital to the aquaculture industry for this very reason (49). Microalgae naturally produce omega 3 to serve as an energy source during cell division or unfavorable growth conditions (49). Microalgal strains such as *Phaeodactylum tricornutum* and *Nannochloropsis* have been shown to possess an EPA content of up to 39% of total fatty acids, while some strains such as *Thraustochytrium* and *Schizochytrium limacinum* can contain a DHA percentage of between 30 and 40% of total fatty acids (49). Finally, increased production of omega 3’s can be induced with either various environmental stressors or metabolic engineering (49).

Despite the increasing use of algae in food products, only a small percentage of these products have high algae caloric and nutritional content, due to their overall low algae content. However, algae themselves still have a markedly higher protein production potential than traditional crops, with a 4- to 15-fold increase in protein productivity per acre over soybeans, wheat, and rice. Algae also have an advantage in that they can produce a broader range of amino acids; especially essential amino acids that are required in the human diet (18, 52). This makes the incorporation of algae into food directly applicable to increasing the nutritional quality of human food products and is further optimized by selecting algae with a complete nutrition profile. Though not many currently studied strains fit this profile, the road to the development of algae with a diverse suite of nutritional components is supported by recent studies that increased both the biomass and nutritional content of algae through a combination of synthetic biology, breeding, and mutagenesis (18, 53).

### 2.5 Enhancement of pigments and other nutritional molecules

Complementing algae’s complete nutritional profile, is their production and accumulation of additional nutrients such as essential vitamins and minerals. Algae have a naturally high content of carotenoids–a family of red, orange, and yellow pigments that are converted to vitamin A within the human body. Aside from their ability to capture light, carotenoids are also useful scavengers of reactive oxygen species (54, 55). This gives carotenoids useful antioxidant properties, which are attributed with lowering the risks of cardiovascular and macular-degenerative diseases, and may possibly mitigate risk of certain cancers (56–59). Since carotenoids are lipid-soluble vitamins, many plants which also synthesize them in copious amounts must be paired with an additional source of fat in order to make the vitamins bioavailable (60). Due to their previously discussed lipid rich content, algae inherently have properties that allow them to serve as an ideal method of delivery. Within the scope of industrial applications, it may be of interest that colorful fish such as wild salmon get their color from the carotenoids that travel up the biological food chain. Farmed salmon are given this color by artificially incorporating astaxanthin, a carotenoid, into their diet (18). As opposed to a synthetic production approach, demonstrated improvements in algae carotenoid production may position them as a more desirable source of carotenoids for farmed fish, which serve as a proxy for supplementing algae into the human diet (61). Regarding human health, astaxanthin exhibits strong antioxidant activity, thereby giving the carotenoid anti-inflammatory and UV light protecting properties (62).

Lutein is another essential carotenoid that is found in human food coloring, and coloring in poultry and fish (63). Because of high carotenoid content in the human eye, several studies have been conducted to research lutein as a nutraceutical for problems regarding vision and the structure of the eye (64). Epidemiological studies have reported that lutein in diet supplements could prevent the development of age-related macular degeneration (AMD, blindness in old-aged people) and cataracts (65). The current conventional commercial source of lutein is marigold oleoresin flowers; however, the potential for high lutein production in microalgae can pave the path toward a more efficient commercial source (65). Within a 7-day period, lutein production and productivity via both heterotrophic and photoautotrophic cultivation, respectively, of *Auxenochlorella protothecoides*, a green alga, were 34 mg/L and 12 mg/L/day, which resulted in a 10-fold increase in lutein content when compared to marigolds (66). All these properties contributes to make microalgae an ideal candidate for meeting global dietary needs.

## 3 Resource use for algae cultivation and comparison to traditional crops

### 3.1 Nutrient usage for algae cultivation

In traditional agriculture, as well as in algae farming, nutrient use is a major factor when it comes to the sustainability of food production. Both algae and conventional crops require a mixture of nitrogen, phosphorous, and potassium for growth, which can be provided in the form of fertilizers. Most synthetic nitrogen fertilizers are produced from atmospheric nitrogen through the Haber-Bosch process (35). Unfortunately, this process is highly energy-intensive and also poses environmental challenges due to its dependence on fossil fuels and high CO_2_ emissions (67). Phosphorus is in limited supply world-wide, and can only be obtained via mining, which in itself causes environmental damage (68). Nitrate and phosphate salts are common sources of nitrogen and phosphorus used in media for growing algae. Assuming that all nutrients are efficiently absorbed by the crop, algae were found to require a total of 40–90 kg nitrogen and 3–15 kg of phosphorus per ton of algae biomass (68). These nutrients may come from synthetic fertilizers or other sources, such as waste streams (6).

Corn, on the other hand, is the crop that requires the most nitrogen fertilization in the USA, and the rate at which nitrogen fertilizers are applied is around 170 kg of nitrogen per hectare (69, 70). Corn yields vary by region, averaging at 6 tons (5.67 metric tons) per hectare worldwide and 12 tons (10.8 metric tons) per hectare in the USA, alone (71, 72)This corresponds to approximately 14–27 kg of nitrogen fertilizer per ton of corn. A similar analysis of phosphorus use shows corn uses 6–12 kg of phosphate fertilizer per ton of corn produced (69). This means that for algae cultivation, the use of phosphorous is comparable to that of corn per unit of biomass. As far as nitrogen usage, current algae production requires more usage of nitrogen than corn by biomass. However, algae crops can contain up to 70% protein content whereas corn averages at about 3% protein content according to the U.S. Department of Agriculture (USDA) (70). This means algae production could more efficiently use nitrogen per unit protein. Developing systems that take advantage of the nutrients already present in wastewater can provide a platform to grow algae that does not heavily depend on the Haber-Bosch process or phosphorus mining. Furthermore, the additional cost of adding nitrogen could potentially be mitigated by co-culturing microalgae with cyanobacterial stains that are capable of their own nitrogen fixation (73).

Micronutrients like calcium, iron, and magnesium are also needed in smaller amounts to cultivate algae. Agricultural, industrial, and animal wastewaters also contain these micronutrients, as do some sea waters (68). The issue with using wastewater as a source of these helpful nutrients it that pathogens or heavy metals can also be present (depending on the type of waste streams), which would be a cause for concern when growing algae for food and feed. Adding treatment processes such as aerobic digestion or alkaline treatment between the collection of wastewaters and the cultivation of algae biomass has been proposed as a potential solution to these challenges (74). Seawater is another alternative source to freshwater growth that can be utilized in order to grow algae for food. Although it is low in iron, seawater contains high levels of magnesium and calcium, and is already being used as tool to increase the biomass and lipid culture of various algal species for biodiesel production-the methods of which can be expanded to algae produced expressly for food production (75).

### 3.2 Algae cultivation methods and resource use

**FIGURE 2:**
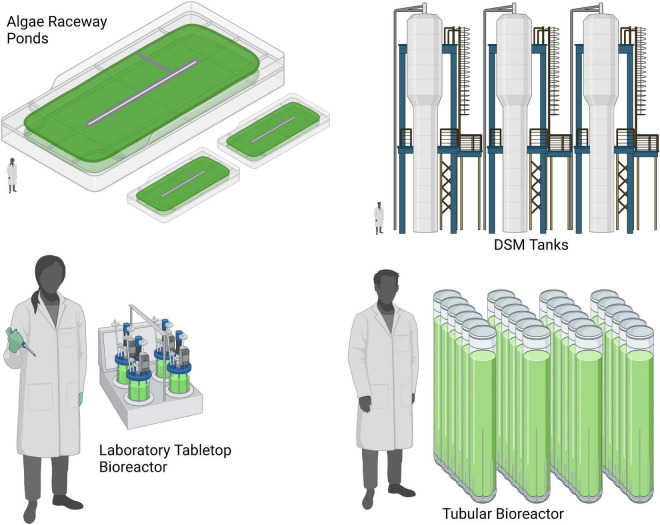
Visual comparison of various algae cultivation methods (Created with Biorender.com).

The resource consumption associated with growing algae for food is highly variable depending on the systems in which the algae are grown and harvested (76, 77). The three most prominent growth systems are open-air ponds, photobioreactors, and heterotrophic bioreactors (Figure 2). Both open ponds and photobioreactors utilize photosynthesis as the primary input of energy, while heterotrophic tanks utilize reduced carbon as the primary energy input. Independent of the system used, all methods of algae cultivation require some basic energy expenditures for mixing and gas exchange, as well as basic nutrient supplies in the form of nitrogen, phosphate, potassium, and other inorganic minerals (78).

Heterotrophic growth in bioreactors is currently used for large-scale commercial algae production (79, 80). These systems have one enormous advantage in that extremely high cell density can be achieved: often orders of magnitude higher than under photosynthetic growth conditions (81). These high cell densities result in a much lower spatial footprint per ton of biomass produced, and both harvesting and downstream costs can be significantly reduced (81). Bioreactors can also reduce contamination but require significantly higher capital expenditures as well as significant operating expenses to maintain adequate control over mixing, gas exchange, and temperature. This can be several orders of magnitude higher than open ponds and photobioreactors (82). Heterotrophic growth also requires the addition of a carbon source, which also increases the cost of culture media and drives the cost of these combined inputs much higher than that of photosynthetic-based systems (83). For this reason, heterotrophic growth is exclusively used for the cultivation of high-value products like omega-3 fatty acids and other human nutraceuticals (76).

### 3.3 Energy usage for cultivation and processing of algae

Maintenance of the algal cultures can require various energy inputs. The main processes that require energy inputs for production are: culture mixing for aeration, maintaining culture temperature, harvesting (dewatering), and processing into the required product format (82). Additional energy requirements can differ greatly depending on whether photosynthetic or heterotrophic growth are being implemented. Photosynthetic growth that occurs in a bioreactor will require a constant input of light, which can only come from a constantly operating artificial light source. This is in contrast to heterotrophic growth which does not require this constant light input, but still requires consistent mixing to provide the proper amount of aeration and the added carbon source (84). Regardless of the cultivation system used, mixing, harvesting, and processing generally need to be run on the entire volume of the culture, regardless of cell density. Thus, energy use per kilogram of biomass is greatly impacted by the culture density. This highlights the importance of being able to grow algae at high biomass densities, regardless of the system used for production.

In order to assess the environmental impacts of energy use for growing algae as a food source, it is necessary to compare the energy demands of cultivating algae compared to other crops like soy and corn. Soybeans use 3.67 GJ of energy to produce 1 ton of soybeans, which is equivalent to 1.12 kWh per kg of soybeans (85). Corn requires slightly less energy to produce, using 0.738 kWh per kg of corn (85). Based on one NREL study, algae grown in open ponds had a total farm power demand of 0.536 kWh per kg of ash free dry weight (AFDW) of algae biomass (86). Based on these numbers, algae seem to be competitive with traditional crops when it comes to energy use. However, in addition to the energy demands of growing algae, there are energy costs associated with the harvesting and dewatering processes.

For harvesting, centrifugation can be energy intensive, while filtration or flocculation are much less energy intensive (87). The harvesting processes that generally separate algal biomass from the water it is grown in are highly energy intensive and have been cited as major limitations to the sustainable implementation of algae as a biofuel, which is a downside that is likely to translate to algae harvested for food (88). The energy demands associated with harvesting algae from open ponds have been estimated to be 0.2–5 kWh per kg of algae biomass using traditional systems (44). This indicates that the energy demands of harvesting algae, particularly algae from dilute cultures growing in open ponds, could be up to 10 times the energy cost of growing algal biomass. In order to keep the resource demands of growing algae for food low, it will be important to prioritize energy efficient harvesting methods and high-density cultures, and newer gravity filtration systems appear to be capable of greatly reducing this energy use (89).

One life cycle analysis using a weighted nutrient density metric found that “Spirulina tablets” tend to be less of an environmental impact than milk production when it came to factors like GHG emissions and fossil fuel depletion (90). This analysis included the examination of protein content as well as micronutrients such as beta-carotene. The same research also predicted that fossil fuel usage for algae cultivation will decrease by half in the next 5–10 years, based on improvements in the cultivation process. The improvements described mainly involve more efficient use of synthetic fertilizers, which can be energy intensive to produce. Under these improved conditions, the GHG emissions and fossil fuel usage associated with cultivating algae for food would be similar to that of tofu products, which have become a popular protein alternative (90). There are currently very few studies examining the resource use associated with growing other promising algae species for food. Further studies are necessary to determine the potential of species like *Chlamydomonas reinhardtii*, *Nannochloropsis*, and *Aurantiochytrium*, all of which have been proposed as potential food sources (1, 91).

### 3.4 Land usage for algae cultivation

Land is another important resource to examine when assessing the viability of various algae species and cultivation methods for food production. First generation biofuel crops, like corn and soy, compete with food for arable land, thus leading to problems with food supply and increased price. Early interest in land use associated with growing algae came from studies of algae as a second-generation biofuel (92). One study found that algae could produce one GJ of energy per year using 2–13 square meters of land. This was significantly lower than the land use associated with first generation biofuel crops, with corn grain needing 133 square meters for 1 GJ of energy in the form of bioethanol and soybeans used 689 square meters for 1 GJ of energy in the form of biodiesel (93). Other studies examining resource use connected to growing algae for bioenergy had slightly different results, but still found traditional crops to be significantly more land use intensive per unit of energy produced (94).

Many papers have extended these findings to show that using algae for food will reduce the land needed to feed the growing population (18, 52, 95). One life cycle assessment looked at land use per kg of protein powder in order to assess algae more directly as a food commodity rather than a fuel. Land use associated with growing *Chlorella* and *Spirulina* was found to be similar to crops like soybeans and peas, and lower than the amount of land needed for animal-based proteins such as eggs (77). Similar to the case of energy use, algae was found to be less resource-intensive than animal products, but had a similar requirement to crop plants. However, many discrepancies remain between the results of former biofuel studies and more recent work examining algae as a source of food and feed. This could be related to the different functional units used in these studies (GJ of energy as opposed to kg of protein). Therefore, more work is needed to examine algae cultivation using food related metrics such as protein or nutrient production. Two such differences were found in the results of Singh et al. (93) and Smetana et al. (77) which may also be explained by the way various studies define “land use.” Work that differentiates between arable and non-arable land shows how growing algae can be far more efficient than growing conventional crops (93, 94). One study using food-related metrics while taking into account the use of non-arable land, found that replacing 50% of the total 2011 European market for oils and proteins with sources from the diatom *P. tricornutum* would only require 0.5–1.4% of non-arable land available, depending on the cultivation method used (7).

### 3.5 Water usage for algae cultivation

When cultivating algae for biofuels, algae can be cultivated in degraded and nutrient-dense eutrophicated water (96). One study demonstrated the growth of *Chlamydomonas polypyrenoideum* on wastewater from the dairy industry (97). To reduce the freshwater depletion associated with growing algae as food, processes that take advantage of algae’s ability to grow in brackish or seawater coupled with water recycling following algae harvesting will need to be utilized. Spirulina (*Arthrospira platensis*), a cyanobacteria, is already approved for food use, and is normally cultivated in a mix of freshwater and seawater (98). This decreases the dependency of algae cultivation on freshwater alone and helps reduce the risk of contamination by increasing the salinity of the culture. Meanwhile, many marine cyanobacteria can be readily grown in conditions of high salinity since their natural habitat consists of seawater (99). Despite the decrease in their requirement for freshwater, algae still require a water source to grow in. Studies have shown that for every kilogram of biomass that is produced, nearly one metric ton of water is utilized (100). This would mean that algae utilize approximately 1,000 m^3^ per ton of biomass, which is still less than that of prominent traditional crops. Soybeans and lentils, for example, consume 2,145 and 5,874 m^3^/ton, respectively (101). Although the results of these comparisons may seem counterintuitive, it demonstrates that algae cultivation requires far less water than traditional crops, per ton of biomass produced.

Temperature control is also a key requirement to achieve optimal growth. The temperature is naturally regulated in open ponds by evaporative cooling; however, this is detrimental to water usage in warm, arid climates. While closed system photobioreactors reduce direct water input, the lack of natural cooling invariably translates to the need for artificial external cooling. For tubular bioreactors, this is often achieved by misting the enclosure with water for external evaporation. This cooling technique does not necessarily result in an overall reduction of water usage over open ponds, but in some instances allows for the use of less refined water sources depending on the algae being grown (16). In terms of capital expenses, open-air systems have a redeeming quality in that they require much lower initial capital expenditure to build, which is a favorable characteristic when growing algae for relatively inexpensive commodities like food (102). Furthermore, open-air systems can often be built on barren, inhospitable land such as the arid desert (103).

## 4 Enhancement of desirable traits in algae

### 4.1 Breeding as a tool for trait enhancement

It takes several generations, and sometimes decades, for crop breeders to improve crops to become more nutritionally and socio-economically valuable (18). To this day, all major crops continue to undergo constant optimization through breeding methods. Breeding programs that do execute continuous optimization of quantitatively inherited traits are crucial to meeting global demands and adapting agriculture to climate change (104). For instance, the Brazilian Agricultural Research Corporation (Embrapa) develops improved crop strains to provide Brazil with food security. In the USA, the USDA has several agencies that are responsible for agricultural biotechnology, such as NIFA, ARS, and GIPSA. For microalgae, the breeding process to identify improved traits can take as little as a few weeks or months (105). Since microalgae are microscopic organisms and can be mated, grown, and selected in just a few weeks, millions of cells can be mated and undergo selection in a single flask. This makes microalgae an almost ideal candidate for breeding and selection to identify strains capable of mass production of nutritious food; however, algae breeding has only been used sparingly to improve agriculturally important traits (Table 2) (105–107).

**TABLE 2:** Summary of breeding methods and genetic tools used to enhance the production of desirable products.

Breeding/Genetic tools	Types of mutations	Screening techniques	Products	Species	References
Carbon beam irradiation mutagenic breeding	Insertions/deletions in an isoamylase-type starch DBE gene ISA1 were determined in mutant KOR1	Light/dark conditioned FACS screening	Carotenoid, lutein, β-carotene, chlorophyll	*Chlamydomonas* sp.	(54)
Three-stage UV irradiation mutagenesis breeding	No specific type of mutation mentioned	Measuring cell dry weights, plotting growth curves, and plating	Astaxanthin	*Haematococcus pluvialis*	(11)
Heavy-ion irradiation (HII) mutagenesis breeding	Higher mutation rates determined by sharper HII peak (Bragg peak); mutations caused by HII mutagenesis are more stable due to a high linear energy density (64)	Total protein content was analyzed using the Kjeldahl nitrogen determination method; amino acids were measured by the amino acid analyzer (A300; membraPure, Berlin, Germany)	Proteins, amino acids	*Chlorella pyrenoidosa*	(108, 11)
Heavy-ion irradiation (HII) mutagenesis breeding	No specific type of mutation mentioned	Mutants were selected by isolation with FACS and BODIPY staining	Lipids, paramylon	*Euglena gracilis*	(113)
Polyethylene glycol (PEG)-mediated chemical protoplast fusion; UV mutagenesis; ethyl methane sulfonate; compound mutagenesis	No specific type of mutation mentioned	Soluble protein was determined by reading absorbance values at 590 nm; total protein was measured using the Kjeldahl nitrogen method	Proteins, amino acids	*Chlorella sorokiniana*	(133, 11)
Transformation: Microalgae	Exogenous DNA insertion	Colony PCR	Proteins/Amino acids phosphite	*Chlamydomonas reinhardtii*	(138, 140)
Transformation: Cyanobacteria	Homologous recombination	PCR	Lipids	*Synechocystis* sp. PCC 6803	(168)
CRISPR	Site-directed modifications: nuclear inactivation, single point-mutations, gene insertion/deletion	PCR	Proteins/Amino acids	*Chlamydomonas reinhardtii*	(147, 145)

### 4.2 Growth conditions and strain improvement in microalgae

Genotype and growth environment are the underlying factors that affect the phenotype of algal strains, and each of these factors can be altered to improve the phenotype. Traditional breeding, mutagenesis, and other genetic engineering techniques fall under methods to improve genotype (11, 54, 108). Media composition, cultivation systems, biotic and abiotic factors, and other growth conditions fall under environmental factors to optimize phenotype. By combining strain improvement methods and environmental conditions, superior strains and processes can be developed to meet a variety of global demands. Furthermore, breeding strains of microalgae to take advantage of specific environments, such as high temperature, high salt or high pH, can reduce predators and pathogens, and yield even higher biomass productivity and greater nutritional value.

This can be achieved by optimizing the media composition, cultivation system, biotic and abiotic factors, and other growth conditions, and then selecting for strains that thrive under these specific conditions (18). Isolating and breeding novel strains capable of high productivity in extreme conditions, such as high salt (i.e., brackish water) and high pH (>10), is a key component to large scale production. Growing these extremophiles in these harsh environments decreases the unfavorable microbial diversity, reduces culture crashes caused by microbial contamination and predators, and increases stress tolerance while still producing lipid, carotenoid, protein, and carbohydrate amounts suitable for meeting food demands on a global scale (109).

One experimental study focused on the cultivation of *Chlorella sorokiniana* strain SLA-04, a green microalga, in extreme alkaline pH conditions and discovered that the lipid productivity and yields of the strain grown in extremophilic conditions were comparable to that of strains grown under normal conditions (i.e., algae that grow between pH 6.5 and 7.5) (109). The biomass productivity of SLA-04 in high pH (>10) media indoors and outdoors were 42 ± 4.1 and 74 ± 2.1 mg × L^–1^Day^–1^, respectively. The lipid content and lipid productivity in mixotrophic conditions were 36.7% (w/w) and 0.08 g × L^–1^Day^–1^, respectively. Comparably, the lipid content and lipid productivity of SLA-04 were higher than that of other *Chlorella* strains (*Chlorella* sp., *C. vulgaris*, and *Chlorella minutissima*) that were previously reported in the literature (109). The authors claim that in a more buffered alkaline environment, it could be possible to maintain a high pH during mixotrophic cultivation, which would allow for glucose supplementation that would result in even higher lipid yields and lipid productivity.

In addition to high pH conditions, high salt conditions can also play a role in increasing the nutritional content of algae. For instance, *Amphora* sp. was cultivated under hypersaline conditions and was capable of producing notable chlorophyll a, carotenoid, and fatty acid content (110). *Amphora* sp. is a diatom, or single-cell, eukaryotic microalgae. Diatoms have a variety of biotechnological applications, including waste degradation, biomineralization, oil exploration, forensic examination, and environmental indication (111). *Amphora* sp. exhibits high levels of carotenoids, specifically β-carotene and xanthophylls (110). Although the lipid and protein content of *Amphora* sp. under hypersaline conditions was lower than the published values of other strains of *Amphora* sp., the chlorophyll content was almost 5% of the dry cell weight (DCW) and the carotenoid content was 1.083% of the DCW (110). The C16 fatty acids (C16:0, C16:1, C16:2, and C16:3) represented more than 75% of total fatty acids. Palmitic acid (C16:0) and palmitoleic acid (C16:1) accounted together for more than 72.51% of fatty acids (110).

### 4.3 Mutagenesis using various mutagens and phenotype selection

**FIGURE 3:**
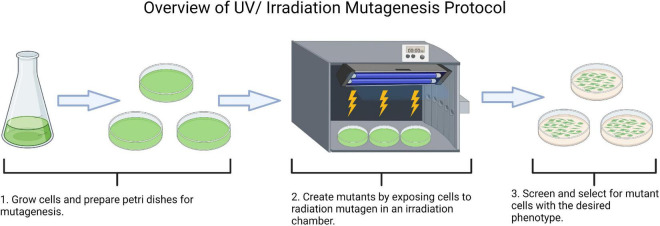
Irradiation (UV) mutation, screening, and selection of microalgae cells (Created with Biorender.com).

Mutagenesis used as a strain modification tool for food applications has the advantage of not being considered a method that generates genetically modified organisms (GMOs) since it does not introduce any foreign genetic material into the cell (112). This process is still currently in use to increase lipid, starch, pigment, protein, and other molecular content in algae (11, 54, 108, 113). Mutagenesis is the act of using chemical mutagens or physical irradiation to expand genetic variation by generating alterations in the genetic code of an organism through mutations in genomic DNA (114). An overview of UV or irradiation mutagenesis is outlined in Figure 3.

With mutagenesis, it is possible to generate vast biological diversity, and you don’t need to know which genes need to be modified, or how they need to be modified, which can be incredibly complex. This is actually a very important tool, because you do not need to understand anything about the underlying genetics, just have a good screening/selection method. Some chemical mutagens are alkylating agents, such as ethyl methanesulfonate, *N*-methyl-*N*-nitrosourea, and azides such as sodium azide (115–118). Physical mutagens consist of electromagnetic radiation [gamma rays, X rays, ultraviolet light] and particle radiation (fast and thermal neutrons, beta, and alpha particles) (119, 120). In the biofuel industry, mutagenesis and selection have been used extensively to identify strains with increased lipid and biomass content. For example, two *Desmodesmus* sp. S81 and G41 (a microalgae) were mutagenized with EMS treatment, resulting in mutants S5 and G3 with higher biomass yield and lipid content (118). The biomass yield for mutants S5 and G3 were 45 and 20% higher than the wild strains, respectively. The lipid content of S5 and G3 were 48 and 46% higher than that of the parent strains (118). Not only is mutagenesis used for increasing biodiesel production, but it can also be used to develop algal strains with higher nutritional content.

### 4.4 Enhancement of carotenoid traits using mutagenesis

One sought-after nutrient that is found in algae is astaxanthin: a carotenoid-based, antioxidant pigment that has been shown to exhibit anticancer, anti-inflammatory, and cardioprotective activity (121). In aquaculture, astaxanthin is essential to develop the orange-red color of farm-raised salmon and shrimp (122). An experimental study done by Kobayashi et al. (123) used chemical EMS and UV mutagenesis to increase astaxanthin content in algae. Based on the cell cycle of *Haematococcus pluvialis*, an algal species that synthesizes high amounts of astaxanthin, a three-stage mutagenesis and selection method was conducted to enhance astaxanthin production. This method consisted of high biomass selection, high photosynthetic activity selection, and high yield astaxanthin selection. First, UV irradiation mutagenesis was employed to increase biomass. The high biomass phenotype was selected during the cells’ green flagellate period. Then, UV and ethyl methane sulfonate (115) mutagenesis were implemented to produce high photosynthetic activity strains, which was inferred from chlorophyll content measurements. Lastly, the selected high biomass and high activity mutants were spread onto agar plates containing diphenylamine (DPA): an astaxanthin synthesis inhibitor. The high yield astaxanthin strain was selected by the change in color of colonies (green to red) on agar plates containing DPA. In *H. pluvialis*, β-carotene is used in the biosynthesis of astaxanthin; and DPA regulates the synthesis of carotene (123). The cells that were still strongly capable of synthesizing astaxanthin in the presence of DPA turned red and were therefore selected. This process generated a mutant with 1.7-fold higher astaxanthin content than that of the wild type (11).

High-throughput screening tools to identify improved traits, such as high carotenoid content, are best if they can be performed on living cells. Unfortunately, this type of analysis is not available for most metabolites. For these particular cases, metabolomics studies can provide a comprehensive understanding of cellular metabolites in organisms, but at present can only be run on populations of cells following extraction of the metabolites. For example, dynamic metabolic profiling and transcription analysis revealed the details of starch-to-lipid biosynthesis switching in *Chlamydomonas* species. This analysis successfully identified that the metabolic rate-limiting step was the conversion from pyruvate to Acetyl-CoA and identified a potential target for metabolic engineering to improve lipid accumulation (54, 124). However, there is currently no way to screen individual cells or colonies for alterations of genes that might impact accumulation of a specific molecule. The only exception to this is when a mutation happens to impact the production of a fluorescent molecule, but that usually requires prior implementation of a fluorescent reporter gene which can then allow individuals to be sorted by expression levels through FACS (18). This is where the usefulness of GMOs will come into play. Knowing the exact location and modifications made to a particular gene will streamline the screening process and increase the overall efficiency of identifying beneficial mutations. However, in order to achieve these types of directed mutations, an assembled and annotated genome is required (125).

A combination of using a physical mutagen and selection have been also used to increase carotenoid content in *Chlamydomonas* (54). The mutations yielded a novel lipid-rich mutant KOR1 with lipid content that was a 2-fold increase relative to that of the parent strain. Analysis of the mutant indicated that isoamylase-type starch debranching enzyme efficiency enhances the degradation of carbohydrates for repartitioning of carbon resources into lipids and carotenoids (10). Additionally, lutein, β-carotene, and chlorophyll content increased in the mutant strain compared to the parent strain. The culmination of these changes enhanced the nutritional value of this strain (121).

### 4.5 Enhancement of protein and carbohydrate traits using mutagenesis

Protein content is another important nutritional characteristic of algae that can be improved upon by utilizing mutagenesis and screening. To increase protein productivity, heavy-ion irradiation (HII) mutagenesis was implemented to generate mutants that were later screened for increased biomass and protein production (108). Compared to traditional mutagenesis techniques, there was a higher mutation rate when HII mutagenesis was performed, which ultimately generated a mutant K05 with a biomass and protein yield which is 30 and 15% higher compared to the parent strain, respectively. This phenotype could be further enhanced by utilizing a low-cost sweet sorghum juice (SSJ), for the heterotrophic production of this *Chlorella* strain. Both the biomass production and chlorophyll content were both higher in this medium. Furthermore, the content of essential amino acids in the mutant K05 in the SSJ medium was higher than wild type, demonstrating that optimizing the environmental conditions coupled with genotype alteration, were the underlying factors to making the K05 mutant a feasible functional food (108).

Mutagenesis has also been identified as a successful approach to generating oil-rich green microalgae *Euglena gracilis* mutants. *E. gracilis* is known to accumulate paramylon as a reserve polysaccharide in response to nitrogen deficiency or heterotrophic carbon sources. Paramylon is a carbohydrate similar to starch and has been reported to play a beneficial role in human health (126). The study induced mutations with Fe-ion irradiation in the wild type strain, stained the intracellular lipids with boron-dipyrromethene (BODIPY) dye, and used FACS-based isolation of the top 0.5% lipid-rich cells with high viability. This method yielded an *E. gracilis* mutant with about 1.4 times higher lipid content than the wild type (113).

### 4.6 Enhancements for organoleptic traits

Not only can algae be selected for higher nutritional value, but they can also be selected to have more appealing organoleptic traits, such as taste, color, odor, and texture. The direct use of algae biomass as food is currently hampered by some perceived unpleasant organoleptic properties: primarily its green color and fish-like smell and taste (127). Visual cues have a direct effect on the consumer’s acceptability of foods and their perception of taste, odor, and flavor of the food (128, 129). Therefore, creating more pleasant organoleptic traits in algae is crucial to increase social acceptance for the consumption of algae as either food or dietary supplements. For instance, an experimental study developed two chlorophyll-deficient mutants of *C. vulgaris*: a yellow-colored MT01 strain and a white-colored MT02 strain, by chemically induced random mutagenesis. The yellow MT01 mutant exhibited a 30% increase in protein content compared to the wildtype, and the white MT02 mutant exhibited a 60% increase. Since these mutants have both higher protein and lower chlorophyll content, they are likely candidates for the development of novel food and dietary supplements that could be more visually appealing to consumers (112).

Organoleptic traits are subjective qualities; and although the aforementioned study stated that green food products usually come with low sensorial acceptance by the consumer, this acceptance can vary from consumer to consumer. Because the organoleptic traits of algae are quantitative traits, it is challenging to identify strains possessing all of the favorable traits that an ideal algae strain might have. Thus, new methods for high-throughput screening of organoleptic traits will ultimately need to be developed for this field to advance more rapidly.

## 5 Methods for targeted enhancement of desirable traits

### 5.1 Protoplast fusion

For many algae, there is presently no method to induce cells to mate. In these cases, protoplast fusion is another technique that can be used to introduce genetic variation. Protoplast fusion is the process of fusing together two distinct species, strains, or mutants, to form a new hybrid containing characteristics of both (130). In this process, two haploid cells are fused using either chemical mediated fusion with polyethylene glycol (PEG) or electrofusion using high voltage (131, 132). For example, the PEG fusion method was used to fuse a high-protein mutant (H10) with a fast-growing mutant (Z13) to produce a mutant that displayed increased protein and amino acid content, as well as rapid growth. The total protein content of one of the fusions R7 (67.16%) was 8.89 and 10.25% higher than that of the original strains H10 and Z13, respectively (133). Although the fibrous cell wall of *C. sorokiniana* (a microalgae) poses a challenge for the preparation of protoplast fusion, different enzyme combinations and concentration treatments can be implemented for effective cell disruption (133, 134).

In addition to improving the protein and amino acid profile of algae, fatty acid content can also be enhanced by implementing protoplast fusion. Using the PEG fusion method, the fatty acid-secreting chrysophyte *Ochromonas danica* (microalgae, aka: “golden algae”) and the astaxanthin-producing chlorophyte *H. pluvialis* were fused. To confirm hybridization of the two strains, the fatty acid profile of the fused cells was analyzed. After protoplast fusion, the characteristic fatty acids of *H. pluvialis* (C16:0 and C18:3n-6) and *O. danica* (C16:2 and C24:0) were exhibited in the resulting cell line. Additionally, the hybrid resembled the morphology of *H. pluvialis* and acquired the green color of *O. danica*, demonstrating that there was a transfer of genetic information into the new strain (131).

### 5.2 Genetic transformation

Genetic “transformations” are molecular processes that insert or modify the genetic makeup of a particular organism, and efficient transformation methods are available for many algal species (10, 135). Algae can be transformed, and their progeny grown in a fraction of the time that it takes for most plant species. Not only does the generation time of a terrestrial plant take longer, but many plants can only be transformed using an agrobacterium transformation. This is a process in which the bacteria transform the plant with plasmid DNA via horizontal gene transfer, which is a time-consuming process (136). For algal transformations, it is possible to directly introduce DNA into the algal cells on coated metal particles, or by electroporation which uses electric current to compromise the cell membrane and allow entry of DNA into the cell. Electroporation transformations achieving efficiencies of up to 2–6 × 10^3^ transformants per microgram of exogenous DNA have been reported (137, 138). Homologous recombination of transgenes into the native genome can be hugely beneficial for creating transgenic strains. However, current genetic transformations for most nuclear genes in algae rely on random integration of exogenous DNA into the genome. This form of insertion is viable, but not ideal due to the variety of off-target effects (which could impact a multitude of cellular processes) that come with random DNA insertion into a genome.

The presence of three different genomes within a single-celled organism make microalgae a unique candidate for genetic transformations (139). In particular for *C. reinhardtii*, the fully annotated genome (including the nuclear, mitochondrial, and chloroplast genomes) are all amenable to modification by genetic transformation (139). One of many enhancements made via nuclear transformation was the genetic modification of the *C.reinhardtii* nuclear genome by introduction of a gene known as *ptxd*. The resulting transgenic line displayed an improved phenotype in the form of an ability to use a new source of phosphorus. In this study, the modified microalgae could use phosphite (PO_3_^3–^) as a phosphorous source rather than depending solely on phosphate (PO_4_^3–^), its fully oxidized form Sandoval-Vargas et al. (140). A comprehensive overview of methods by which microalgae transformations have changed over the years can be found in Wang et al. (138).

Transformations in cyanobacteria can be very similar to that of non-photosynthetic bacterial strains (99). Natural transformation, conjugation, and electroporation are all methods of transformation which are available for cyanobacteria, but not all transformation strategies can be applied to all strains (141). In many cyanobacteria, exogenous plasmid DNA can be expressed directly since the transcription machinery and plasmid DNA are both able to move freely about the cell. This allows cyanobacteria to have exogenous genes inserted into their genomes, or for the expression of exogenous genes that are carried on replicative plasmid vectors, but not all strains are capable of undergoing both types of gene expression (142).

### 5.3 CRISPR technology in algae

CRISPR technology utilizes machinery that was originally identified as a defense mechanism for bacteria against viral pathogens (143). This system has been modified as a method for precise genetic modifications, and a broad range of CRISPR/Cas-associated proteins have been discovered as this system continues to be characterized (144). CRISPR technology has been applied to algae and makes site-directed genetic modifications possible within these diverse organisms. However, this has not been achieved without some difficulty. Previous issues with implementing CRISPR arose from an inability to express the Cas protein component of CRISPR complexes from expression vectors transformed into microalgae (145). This issue has been circumvented by using electroporation to transiently transform cells with a pre-assembled CRISPR ribonucleoprotein (RNP) complex. This RNP complex consists of a Cas protein that creates double-stranded DNA cuts and a synthetic guide RNA that targets the Cas protein to the desired region within the genome (146). Reports of using both a Cas9 and a Cas12a CRISPR complex have emerged which demonstrate the successful transformation of the microalgae *C. reinhardtii* in order to knock-out nuclear genes (147, 148). CRISPR has also been shown to successfully create gene insertions and single-point mutations in algae (115, 149).

Parallel successes have been made for the genetic engineering of cyanobacteria using CRISPR (150). Like other bacterial species, cyanobacteria (with the exception of *Synechococcus* and *Prochlorococcus*) have their own native CRISPR-Cas systems for defense against viral pathogens (151). In terms of genetic engineering, both Cas9 and Cas12a have been used to make gene deletions in cyanobacteria (151). RNA interference protocols that implement a dCas (aka “dead” Cas) which targets and physically blocks a specific RNA from being synthesized have also been reported (151). Although the current methods are not yet highly efficient, this is a promising start to developing tools that will allow for creating specific genetic modifications in algae. A comprehensive overview of genetic tools such as these has been reviewed by Vavitsas et al. (10) and Kumar et al. (125). A summary of the different breeding methods and genetic tools mentioned in sections 4 and 5 can be found in (Table 2).

## 6 Regulatory challenges of food production and GMO algae

### 6.1 GMOs and algae

While traditional breeding and mutagenesis are effective tools, these are only capable of augmenting genes which can thereby enhance specific characteristics that already exist within a population. As previously stated, the desirable traits of algae include the major dietary components needed for a healthy diet such as lipids, carbohydrates, and proteins. The enhancement and/or addition of these traits can be addressed through the generation of GMOs. The types of organisms that fall under this category vary from region to region. In the USA, GMOs are defined as “an organism produced through genetic modification” by the USDA (152). However, genetic modifications have been further defined as introducing new genetic material into a cell or organism (153). A naturally occurring algae strain that has all of the necessary traits to provide humans with the ideal amount of dietary nutrition has yet to be discovered (Tables 1.1, 1.2). Genetic engineering stands out as the method of choice to supplement the nutritional profile of algae as a superfood, and thus address nutritional deficiencies worldwide (154). Due to the accelerating pace of climate change and its negative impacts on traditional agriculture, having fast and simple genetic modification techniques for biological food sources is crucial. Algae are prime candidates for these modifications as they have already demonstrated fast growth rates, easy methods of genetic engineering and selection, and the culturing and processing techniques obtained from the biofuel industry are easily translatable to food production. One of the biggest concerns is the lack of public trust in GMO foods, as well as a lack of exposure to foods containing algae. Ironically, much of the food Americans consume today is already made with GMO-derived ingredients with “approximately 9 out of every 10 acres of domestic corn, cotton, soybeans, sugar beets, and canola were cultivated using GE (121) seeds…” (152). These numbers indicate that whether they are directly used in food, or added during processing that occurs prior to being consumed, GMOs and their byproducts are still being consumed. New regulations have been developed for the specific labeling of GMO foods despite the fact that they are considered just as safe as non-GMO foods by a combination of the Environmental Protection Agency (EPA), Food and Drug Administration (FDA), and the USDA (155).

### 6.2 Algae that have received GRAS status

Although algae are a relatively new to the American food market, there are many species of algae that have already achieved GRAS status by the FDA (Table 3). The acronym “GRAS” stands for “Generally Recognized as Safe,” and is a status given by the FDA for a specific substance that is safe for human consumption (18). In order to receive GRAS status in the USA, it must first be determined by the FDA whether “the scientific data and information about the use of a substance must be widely known and there must be a consensus among qualified experts that those data and information establish that the substance is safe under the conditions of its intended use” (156). The species of algae that have already been given GRAS status include *A. platensis*, *C. reinhardtii*, *A. protothecoides*, *C. vulgaris*, *Dunaliella bardawil*, *E. gracilis*, *H. pluvialis*, and *Schizochytrium* (18, 157). This is an important step on the path to making algae a reliable food source and should help improve the social acceptance of algae as a future source of human nutrition.

**TABLE 3:** Intended vs. commercial uses of GRAS status algae.

Species	Genus	Intended use	Commercial use	Reference
*Arthrospira platensis*	*Arthrospira*	Food additive (169)	Dermatological products/Cosmetics	(170)
*Chlamydomonas reinhardtii*	*Chlamydomonas*	Dietary proteins (171)	Biofuel production	(172)
*Auxenochlorella protothecoides*	*Auxenochlorella*	Dietary proteins (173)	Biodiesel production	(174)
*Chlorella vulgaris*	*Chlorella*	Food ingredient (175)	Dietary and medicinal supplement	(176)
*Dunaliella bardawil*	*Dunaliella*	Food ingredient (179)	Dietary and medicinal supplement	(180)
*Euglena gracilis*	*Euglena*	Food ingredient (179)	Dietary and medicinal supplement	(180)
*Haematococcus pluvialis*	*Haematococcus*	Food ingredient (181)	Dietary and medicinal supplement	(182)
*Schizochytrium*	*Schizochytrium*	Food ingredient (183)	Food supplements and dairy products	(184)

## 7 Conclusion

Algae have the natural potential to display high biomass productivity, excellent nutritional properties, appealing organoleptic traits, resistance to abiotic and biotic stresses, and the potential for commodity scale production. Algae biomass is a diverse, nutritious, and efficient option for the cultivation and production of new food products. Algae possess several key features that set them apart from conventional crops in terms of sustainability and production of high-value macronutrients. Compared to traditional crops, the percentages of lipids and proteins can be much higher in algae, since they do not require the use of non-edible cellulose for their structural components. As a result, even low biomass production can generate high levels of essential nutrients compared to terrestrial plants.

Current technologies in selection, breeding, and genetic engineering can all contribute to the advancement of nutrient and biomass production in algae. However, there is still much work to be done in order to domesticate algae and obtain strains that exhibit the desirable traits, a process which has been developed in traditional crops plants over millennium. Examples of these traits are rapid growth, resistance to pests and pathogens, and nutritional profiles. There is a current lack of studies that have measured environmental impacts and resource requirements of algae produced for human consumption. However, information gathered from previous studies provided by the biofuel industry can be used as a basis on which these studies can be built. With further research, culturing techniques and production efficiency can be improved upon to create a more environmentally sustainable future with algae as a key nutritional component of people’s diets.

While it has been shown that there has been a slight increase in the transition to alternative protein sources, getting the general public to accept algae as a new, healthy food product, remains a significant challenge. This will mainly depend on different marketing strategies to inform the public of the health benefits these organisms can provide, as well as using breeding and genetic modifications to increase yield and improve the flavor of algae. If the acceptance of GMO algae products continues to be a barrier to creating large-scale demand, the option to produce non-GMO algae at scale still remains. The number of products that can be produced from algae are as diverse as the organisms themselves, which allows algae to be cultivated for these other products while genetically modified algae are debated as a new food source. In either case, algae clearly have the capacity to contribute to the production of healthier and more sustainable food products in the near future.

## Author contributions

CD: contributed to coordinating, writing, tables and figures construction, and editing of original draft. KD: contributed to writing, figures construction, and editing of original draft. KK: contributed to writing, tables and figures construction, and editing of original draft. AK and RM: contributed to writing, tables construction, and editing of original draft. JM, YT-T, and AB: contributed to editing of original draft. SM: contributed to writing and editing of original draft. All authors contributed to the article and approved the submitted version.
